# Weak subjective–facial coherence as a possible emotional coping in older adults

**DOI:** 10.3389/fpsyg.2024.1417609

**Published:** 2024-09-04

**Authors:** Wataru Sato, Akie Saito

**Affiliations:** Psychological Process Research Team, Guardian Robot Project, RIKEN, Kyoto, Japan

**Keywords:** facial expressions, subjective experience, mind-body coherence, older adults, emotional coping

## 1 Introduction

Facial expressions play a critical role in intra-individual emotional functions (Finzi and Rosenthal, 2016) as well as inter-individual social functions (Keltner and Kring, 1998). Facial expressions have been proposed to be more important in late adulthood, given the increased value placed on intimate social relationships (Carstensen, 2006, 2021; Carstensen et al., 1999). In studies examining changes in emotional functioning over the course of adulthood, older adults' facial expressions in response to emotional stimuli have mainly been examined through comparison with those of young adults (e.g., Levenson et al., 1991).

Consistent with the notion of emotional functions of facial expressions, several studies in young adults have reported that the production of facial expressions showed coherence with the subjective experience of emotional feeling (e.g., Mauss et al., 2005; Evers et al., 2014; Sato et al., 2020, 2021; Sato and Kochiyama, 2022). For example, a study measured dynamic ratings of subjective emotional valence (i.e., hedonic tone ranting from positive to negative; Barrett, 2006) and facial electromyography (EMG) activity of the corrugator supercilii (i.e., brow lowering muscle) and zygomatic major (i.e., lip corner-pulling muscle) while participants watched emotional films (Sato et al., 2020). The results showed that the EMG activity of the corrugator supercilii and zygomatic major muscles was negatively and positively associated with the dynamic valence ratings, respectively. These findings are theoretically important because they empirically support the long-lasting idea that subjective emotional experience depends on the perception of bodily responses (James, 1884; Friedman, 2010; Lang, 1994). Furthermore, this issue could be practically important, because a previous study showed that the coherence between subjective emotional experience and bodily responses to emotional stimuli is associated with psychological wellbeing (Brown et al., 2020).

However, the coherence between emotional experience and facial expressions has not been fully examined in older adults. This issue could be interesting because several studies have suggested that older, compared with young, adults have improved emotional wellbeing (Carstensen, 2006, 2021; Charles, 2010; Carstensen et al., 1999; Charles and Piazza, 2023). One may expect that older adults would have stronger subjective–facial emotional coherence than young adults. This paper reviews the research conducted to date on this topic and describes our speculative hypotheses (cf. Currie, 2023).

## 2 Coherence between facial expressions and subjective emotional experience in older adults

Several previous studies have examined either the production of facial expressions or subjective emotional experience in older adults and reported mixed findings (Supplementary Table 1). Specifically, a substantial number of studies showed no age-related differences in facial expression production (Emery and Hess, 2011; Kunz et al., 2008; Lohani and Isaacowitz, 2014; Malatesta et al., 1987; Nangle et al., 2018; Saito et al., 2022; Seider et al., 2011; Tsai et al., 2000; van Reekum et al., 2011), although some studies reported less intense (Kunzmann et al., 2017; Labuschagne et al., 2020; Levenson et al., 1991; Magai et al., 2006; Pedder et al., 2016; Rohr et al., 2017; Saito et al., 2023) or more intense (Magai et al., 2006; Malatesta-Magai et al., 1992; Phillips et al., 2008; Zempelin et al., 2021) facial expressions in older than young adults. Fölster et al. (2014) reviewed the literature and concluded that there are no age-related differences in spontaneous facial expressions. Likewise, studies have reported different results with respect to subjective emotional experience, including comparable (Emery and Hess, 2011; Kunz et al., 2008; Malatesta et al., 1987; Nangle et al., 2018; van Reekum et al., 2011), weaker (Kunzmann et al., 2017; Labuschagne et al., 2020; Levenson et al., 1991; Malatesta-Magai et al., 1992; Saito et al., 2022, 2023; Tsai et al., 2000), or stronger (Labuschagne et al., 2020; Lohani and Isaacowitz, 2014; Magai et al., 2006; Pedder et al., 2016; Phillips et al., 2008; Rohr et al., 2017; Saito et al., 2023; Seider et al., 2011; Zempelin et al., 2021) subjective emotional responses in older adults relative to young adults. In short, the data do not consistently suggest alteration in either facial expressions or subjective emotional experience in older adults. Furthermore, the studies were not specifically concerned with coherence between these emotional responses.

Few studies have compared the coherence of facial expressions with subjective emotional experience between older adults and their young counterparts (Lohani et al., 2018; Saito et al., 2022, 2023) according to our computer-based search of abstract and citation databases of the literature. In Lohani et al. (2018), the researchers presented film clips to induce sad feelings as emotion-eliciting stimuli and measured the dynamic ratings of arousal (i.e., the feeling of activation; Barrett, 2006) and facial EMG of the corrugator supercilii in older and young participants. The results showed no significant age differences in the correlations between the subjective ratings and facial EMG. However, the null finding may be at least partially explained by the ratings measured in the study. Previous studies testing young adults and reported subjective–facial emotional coherence reported the links between the ratings of valence, but not arousal, and facial expressions (Bradley and Lang, 2000).

**Figure 1:**
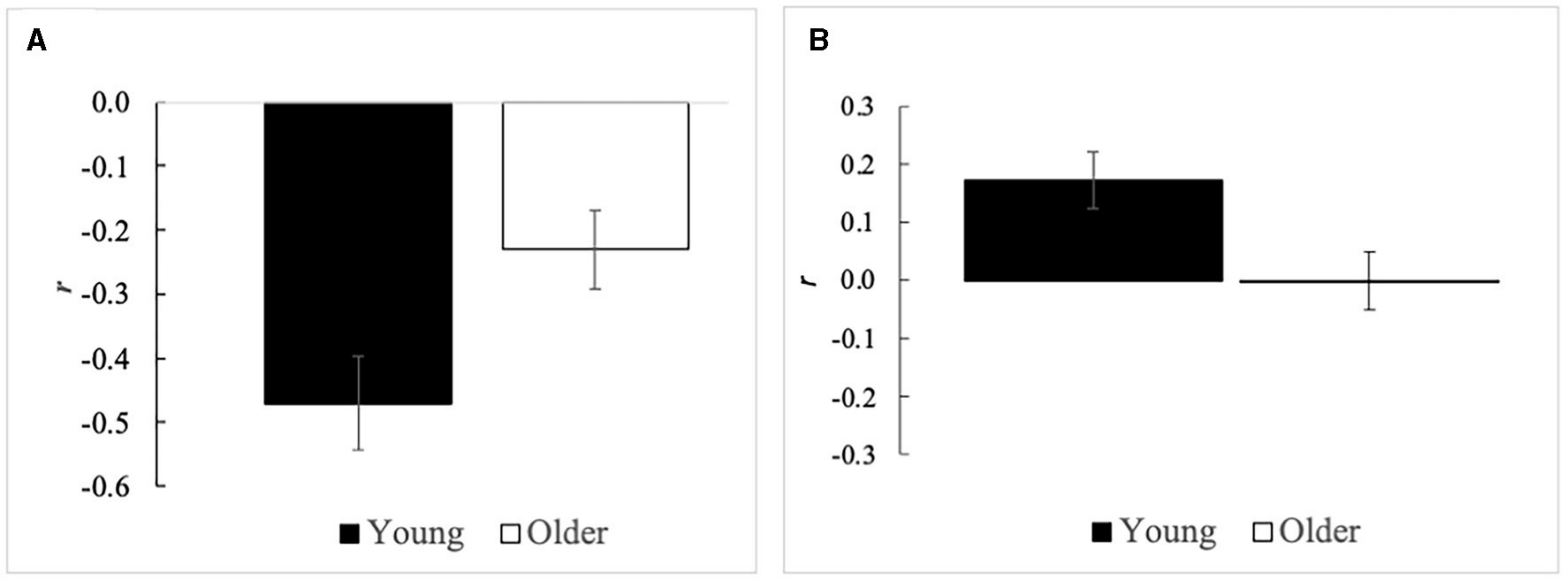
Mean (with standard error) intra-individual correlation coefficients between subjective valence ratings and facial electromyography (EMG) activity in young (black bars) and older (white bars) adults. **(A)** Valence–corrugator (i.e., brow lowering muscle) EMG correlation during emotional film viewing in Saito et al. (2023). **(B)** Valence–masseter (i.e., chewing muscle) EMG correlation during gel-type food consumption in Saito et al. (2022). Both studies showed significantly weaker subjective–facial emotional coherence in older than young adults.

In Saito et al. (2023), five film clips that were categorically labeled as anger, sadness, neutral, contentment, and amusement, were used, and the coherence between dynamic valence ratings and facial EMG of the corrugator supercilia and zygomatic major muscles was assessed by adopting the procedures used in a previous study that demonstrated emotional coherence in young adults (Sato et al., 2020). The researchers compared correlation coefficients between older and young adults and found that the negative correlation between valence ratings and corrugator EMG activity was weaker in older adults (Figure 1A). More specifically, lower valence ratings (i.e., greater unpleasantness) were associated with greater corrugator EMG activity, and the association between brow activity (i.e., frowning, which reflects unpleasant feelings) and unpleasant emotions was weaker in older adults than in young adults, indicating less emotional coherence between subjective experience and brow activity in the former group.

Saito et al. (2022) used food to elicit emotions similarly showed age-related differences in the coherence between facial expressions and subjective experience. In that study, various subjective ratings and facial EMG activity of the corrugator supercilia, zygomatic major, masseter (i.e., chewing muscle), and suprahyoid (i.e., swallowing muscle) were assessed in older adults while they consumed the flavored gel-type foods. Compared with young adults, the coherence of valence and wanting ratings with masseter EMG activity was lower in older adults (Figure 1B).

Taken together, among three studies that tested this topic, the two recent ones (Saito et al., 2022, 2023) suggested that the degree of coherence between subjective experience, specifically its qualitative aspects, and the production of facial expressions in response to emotional stimuli (film clips and food) differs according to age, i.e., is weaker in older adults. As described above, weaker subjective–facial emotional coherence in older adults appears not to be explained by their impairments in subjective emotional experiences or the production of facial expressions.

## 3 Discussion

Given that the mind–body coherence and wellbeing are related (Brown et al., 2020; Mauss et al., 2011), and that older adults tend to report higher levels of wellbeing than people in other stages of life (Carstensen et al., 1999; Carstensen, 2006, 2021), why is it that the coherence between subjective experience and facial expressions is weak in older adults?

We hypothesize that the coherence between the subjective experience and facial expressions may weaken with age as an emotional coping. It has been reported that older adults are better able to cope with emotions (Burr et al., 2021; Charles and Carstensen, 2008; Eldesouky and English, 2018; Scheibe and Blanchard-Fields, 2009; Neubauer et al., 2019; Lohani and Isaacowitz, 2014; Sims et al., 2015; Shiota and Levenson, 2009; however, see Isaacowitz, 2022) and use more emotional coping than young adults (Puente-Martínez et al., 2021). For instance, older adults were more successful than younger adults in terms of both deploying attentional resources (Scheibe et al., 2015; Mikkelsen et al., 2021) and positive reappraisal (Lohani and Isaacowitz, 2014; Shiota and Levenson, 2009). It has also been demonstrated that, rather than implementing response-focused coping, older adults are likely to use antecedent emotional coping (i.e., situation selection) in their daily lives, which is assumed to contribute to their ability to cope with emotions (Sims et al., 2015). Interestingly, several studies have reported that a repressive emotional coping style (Weinberger et al., 1979), which can induce low mind–body coherence (Schwerdtfeger and Kohlmann, 2004) and is traditionally regarded as maladaptive coping (Schwartz, 1990), sometimes serves adaptive functions, such as promoting resilience following extremely aversive events (e.g., Coifman et al., 2007; for a review, see Bonanno, 2005). Likewise, it may be possible that low subjective–facial emotional coherence in older adults has adaptive benefits on their emotional wellbeing.

However, there are several limitations in our speculation. First, few studies have investigated the coherence between facial expressions and subjective emotions in older adults, and those that did used limited methodologies compared with studies that were not specifically concerned with coherence. Thus, future research is warranted to confirm the robustness of the findings. Second, even assuming that older adults have weak subjective–facial emotional coherence, different factors may account for their high levels of wellbeing. The empirical evidence is needed to test the relationship between the subjective–facial emotional coherence and wellbeing in older adults.

In conclusion, studies examining the degree of coherence between subjective experience and facial expressions have demonstrated that older adults showed less coherence of subjective experience with corrugator and masseter EMG activity in response to emotional stimuli (films and food). We speculate that the weak coherence between subjective experience and facial expressions may serve as an emotional coping that is seemingly functionally adaptive for people in late adulthood.
