# Supplementary material for: Weak subjective–facial coherence as a possible emotional coping in older adults
Source: Front Psychol. 2024 Sep 4;15:1417609. doi: 10.3389/fpsyg.2024.1417609 (PMC11408332; doi:10.3389/fpsyg.2024.1417609)
Supplement: Supplementary file 1 [file Data_Sheet_1.PDF]

Supplementary Table 1. Overview of research examining age-related differences in facial expressions and subjective emotional experience.

Authors	Induction method	Facial measurement	Target emotion	Main results
Emery et al., 2011	Photographs	EEB	Positive and negative	Facial expression: -<br>Experience: -
Kunz et al., 2008	Noxious stimulations	FACS	Pain	Facial expression: -<br>Experience: -
Kunzmann. et al., 2017	Relived emotion	EEB	Anger and sadness	Facial expression: ↓<br>Experience: ↓
Labuschagne et al., 2020	Films	EMG	Sadness and amusement	Facial expression: ↓<br>Experience: ↓↑
Levenson et al., 1991	Relived emotion and directed facial action	FACS	Anger, disgust, fear, happiness, sadness, and surprise	Facial expression: ↓<br>Experience: ↓
Lohani & Isaacowitz, 2014	Films	EMG	Sadness	Facial expression: -<br>Experience: ↑
Magai et al., 2006	Relived emotion	MAX	Anger and sadness	Facial expression: ↓↑<br>Experience: ↑
Nangle et al., 2018	Photographs	EMG	Positive, neutral, and negative	Facial expression: -<br>Experience: -
Malatesta et al., 1987	Relived emotion	Decoders' ratings	Affection, anger, fear, happiness, and sadness	Facial expression: -<br>Experience: -
Malatesta-Magai et al., 1992	Structured interview	MAX	Anger	Facial expression: ↑<br>Experience: ↓
Pedder et al., 2016	Photographs	EMG	positive and negative	Facial expression: ↓<br>Experience: ↑
Phillips et al., 2008	Films	Coding of emotional expression	Negative and neutral	Facial expression: ↑<br>Experience: ↑
Rohr et al., 2017	Relived emotion and think-aloud	EEB	Joy and happiness	Facial expression: ↓<br>Experience: ↑
Saito et al., 2022	Food	EMG	Positive and negative	Facial expression: -<br>Experience: ↓
Saito et al., 2023	Films	EMG	Anger, sadness, amusement, neutral, contentment, and amusement	Facial expression: ↓<br>Experience: ↓↑
Seider et al., 2011	Films	FACS	Sadness	Facial expression: -<br>Experience: ↑
Tsai et al., 2000	Films	EEB	Sad and amusement	Facial expression: -<br>Experience: ↓
van Reekum et al., 2011	Photographs	EMG	Positive, neutral, and negative	Facial expression: -<br>Experience: -
Zempelin et al., 2021	Films	FaceReader	Anger, disgust, happiness, and sadness	Facial expression: ↑<br>Experience: ↑

EEB, Emotional Expressive Behavior Coding System (Gross & Levenson, 1993); EMG, electromyography; FACS, Facial Action Coding System (Ekman et al., 1978); MAX, Maximally Discriminative Facial Movement Coding System (Izard, 1979); -, no significant age difference; ↓ , weaker responses in older adults than young adults; ↑ , stronger responses in older than young adults.
